# Shift Work Effects and Pregnancy Outcome: A Historical Cohort Study 

**Published:** 2018-06

**Authors:** Mohammad Hossein Davari, Elham Naghshineh, Mehrdad Mostaghaci, Seyyed Jalil Mirmohammadi, Maryam Bahaloo, Abbas Jafari, Amir Houshang Mehrparvar

**Affiliations:** 1 Industrial Disease Research Center, Shahid Sadoughi University of Medical Sciences, Yazd, Iran; 2 Isfahan University of Medical Sciences, Isfahan, Iran; 3 Health and Environment, Department of Management Planning and Environmental Education, Tehran University, Tehran, Iran

**Keywords:** Pregnancy, Shift Work, Working Women, Pregnancy Complications

## Abstract

**Objective:** Employed mothers face considerable amount of hazards. Especially shift work can impact pregnant women by affecting some hormones. This study was conducted to assess the adverse effects of shift work on pregnancy outcomes.

**Materials and methods:** This historical cohort study was conducted in 2017 in order to assess the effect of shift work on pregnancy outcomes. The subjects were consecutively selected from pregnant women, which referred to Al Zahra and Shahid Beheshti hospitals, Isfahan, Iran for their pregnancy care. The effect of shift work on pregnancy and labor complications (low birth weight, small for gestational age, pre-eclampsia and eclampsia, intra-uterine growth retardation, spontaneous abortion, preterm delivery, excessive bleeding during labor, and type of labor) was assessed. The effect was adjusted for occupation and number of children as well. Data were analyzed by SPSS (ver. 17) usingT-test, chi-Square test and logistic regression analysis.

**Results:** Totally, 429 pregnant women entered the study. There was not a statistically significant difference between morning and shift workers regarding age. It was found that shift work probably increases the incidence of small for gestational age, pre-eclampsia and eclampsia, intra-uterine growth retardation, spontaneous abortion, and preterm delivery, but after adjustment for job and number of children the effect was observed only on preterm delivery.

**Conclusion:** Working in a rapid cycling schedule of shift work may cause an increase in the incidence of preterm delivery in pregnant mothers.

## Introduction

Pregnancy is an important period for women themselves and their families as well. Maintaining physical and mental health during this period could lead to a successful delivery and giving birth to a healthy infant. In this period, women need an environment free from various stresses; this condition may be difficult to provide for employed women. The number of employed women is being increased in the world (1). Due to the social and economic changes in the process of urbanization in Iran, as a developing country, the relationships are changed as well. Due to social and economic changes in the process of urbanization, more job opportunities are opened to women (2).Working pregnant women are at greater risk than other women. The impact of shift work on the body’s hormones brings harm to pregnant women (3).

Many studies have been done about occupational hazards and their complications in pregnancy. But their results are controversial (4). Gabbeet al. pointed out that pregnant women working about 80 hours a week, have a good pregnancy outcome. But a slight increase in working hours can lead to preterm delivery (PTD), pre-eclampsia and intra-uterine growth retardation (IUGR) (5). Increase in physical activities is not significantly associated with high blood pressure, butis considered as a risk factor for pre-eclampsia (6). The risk of being small for gestational age (SGA) is associated with working more than 50 hours a week. Long working hours increase SGA, but has no effect on the incidence PTD (7). Studies have shown that stress in the workplace is associated with increasing PTD, LBW, spontaneous abortion, and IUGR (8, 9). But one study rejected the association between workplace psychological factors and PTD (10). Psychological stress is also a risk factor for such maternal complications as high blood pressure, pre-eclampsia, diabetes and hemorrhage during pregnancy (11). Psychosocial stress resulting from women employment, is an uncertain risk factor for pregnancy complications (12).

If a pregnant woman works less than 20 hours a week, the risk of PTD will not increase, but in night work, the risk may increase (13). LBW and spontaneous abortion are associated with shift work during pregnancy (14). A review article showed that work overtime is associated with an increased risk of harm to human health. There was a poor communication between long working hours and PTD. Psychosocial complications were increased with working overtime, especially long shifts more than 12 hours a day or more than 40 hours a week (15). Night work increases the duration of pregnancy, and reduces intrauterine growth (16). Physical activities during pregnancy has worrying effects on the health status of the mother and the fetus (17).

Due to the inconclusive and contradictory results of the previous studies about the effects of shift work on pregnancy outcomes, this study was conducted to assess the effect of shift work on the incidence of pregnancy complications.

## Materials and methods

This was a cross-sectional study conducted to evaluate the effects of shift work on pregnancy outcomes, which was performed in Isfahan, Iran. Simple sampling method was used to select the participants. All pregnant mothers who were employed before pregnancy referred to the obstetrics unit of Al-Zahra and Shahid Beheshti hospitals during a 6-month period entered the study if they were nulliparous and between 16 and 35 years old. An informed consent was obtained from all participants. Exclusion criteria for this study were as follows: Smoking and alcohol use, history of intra-uterine growth retardation, anti-phospholipid antibody syndrome, fetal infections, and congenital anomalies in previous pregnancies, and also history of acute severe contact with teratogens, maternal diseases such as anemia, vascular and renal diseases, gestational diabetes and diabetes mellitus, obesity, genital infections, placental and umbilical cord abnormalities, hydrops fetalis.

Totally 440pregnant women entered the study who were divided into two groups: morning workers and shift workers. To collect data, three questionnaires were used: 1. demographic data, medical history and habits; 2. occupational data, i.e. job title, and shift work (type of shift work, i.e. night fixed, afternoon fixed, rapid cycling, and slow cycling); 3. complications in the current pregnancy, i.e. spontaneous abortion, PTD, LBW, pre-eclampsia and eclampsia, SGA, excessive bleeding during labor, and type of labor (vaginal or cesarean). Validity of the questionnaires was determined using expert opinion. Its reliability was assessed by Cronbach's alpha coefficient which was 0.80.Questionnaires were filled by two trained midwives. After filling out questionnaires, data were extracted, and analyzed using SPSS Ver. 17 by Student's t-test, chi-Square test and logistic regression analysis. The study was approved by the ethics committee of Shahid Sadoughi University of medical sciences (# 27866).

## Results

Among all participants, 215 mothers were morning workers, and 214 mothers were shift workers. Eleven questionnaires were incomplete, and did not enter the data analysis. Frequency distribution of participants’ jobs is given in table 1.

**Table 1:** Frequency of participants’ jobs

**Job title**	**Number (%)**
Physician, nurse, nursing aid, operating room personnel	160 (31.3)
Teacher (elementary teacher to university professor)	46 (9)
Office workers and industrial workers	122 (23.8)
Others	101 (19.7)

The mean age in the morning and shift workers was 29.2 ± 4.58and 31.7±7.31 years respectively. There were no significant differences between two groups (P value = 0.12).

All of the shift workers were working in a rapid cycling schedule. Frequency of most pregnancy complications was significantly higher in shift workers (Table 2).

Table 3 shows the odds of the effect of shift work on pregnancy complications

## Discussion

Stable circadian rhythm is essential for a successful pregnancy, and has a positive impact on fetal growth (18). Maternal hormones in the natural circadian rhythm regulate the placental performance. On the contrary, disturbed circadian rhythm shifts, negatively affect the developing of the fetus (19). Significant differences were observed between the two groups (morning and shift workers) in the frequency of miscarriage, intrauterine death, pre-eclampsia and eclampsia and premature rupture of the amniotic membrane. But with the adjustment of occupation and the number of children, it was found that shift work had an impact only on PTD.

The results showed that there is a relationship between shift work and pregnancy complications. But only in PTD a significant difference was seen. It means that with adjusting for the effect of occupation, still there was a significant association between shift work and PTD. In other cases, the difference was not significant. It showed that shift work does not affect other pregnancy complications such as miscarriage, fetal death, preeclampsia, eclampsia, SGA, premature rupture of the amniotic membranes, excessive bleeding after birth, and LBW. After adjustment for the number of children, shift work affected miscarriage and PTD. Simultaneously, adjustment for the effect of occupation and the number of children showed that shift work only affects premature labor.

Results of the studies on the effects of shift work on pregnancy complications are different. Bonziniet al. noted that the long working hours and shift work may lead to premature birth, LBW and pre-eclampsia (20). In other studies, the increased risk of PTD, SGA and pre-eclampsia due to rotating shift and work late at night has been pointed out (1, 18). In the present study, it was concluded that shift work during pregnancy leads to an increased risk of miscarriage and PTD that was consistent with the results of the aforementioned studies. There are also other studies with controversial results. Both et al. showed that night work increases child birth weight, and sedentary lifestyle can lead to pregnancy complications (21). Knutsson et al. showed that shift work itself leads to SGA, but is not related to abortion and hypertension (14). A study reported that the risk of PTD, LBW, and SGA as a result of shift work in pregnancy is low (22).

**Table 2:** Frequency of obstetric complications and type of delivery between morning and shift workers

**Pregnancy and labor complications**	**Number (%)**		**p-value**
	**Morning worker**	**Shift worker**	
Caesarean section	127 (59.1)	130 (60.7)	0.212
Spontaneous abortion	27 (12.6)	45 (21)	0.023
Preterm delivery	49 (22.8)	89 (41.6	<0.001
Intrauterine fetal death	1 (0.46)	7 (3.3)	0.007
Pre-eclampsia and eclampsia	17 (7.9)	31 (14.5)	0.031
Small for gestational age	14 (6.5)	25 (11.7)	0.063
Premature rupture of the amniotic membranes	1 (0.46)	4 (1.9)	0.044
Excessive bleeding after delivery	8 (3.7)	6 (2.8)	0.593
Low birth weight	0 (0)	2 (0.9)	0.155

**Table 3:** The odds of the effects of shift work on pregnancy complications (alone and adjusted for occupation and number of children)

**Pregnancy or labor complications**	**Odds Ratio (%95CI** * **)**			
	**Alone**	**Adjusted for ** **occupation**	**Adjusted for ** **number of children**	**Adjusted for occupation ** **and number of children**
Spontaneous abortion	1.84 (1.1-3.1)	1.74 (0.9-3.0)	1.92 (1.1-3.2)	1.84 (1.0-3.0)
Preterm delivery	2.41 (1.5-3.6)	2.28 (1.4-3.6)	2.39 (1.5-3.6)	2.26 (1.4-3.5)
Intrauterine fetal death	7.2 (0.8-59.3)	7.3 (0.8-64.0)	7.0 (0.8-58)	7.5 (0.8-67.5)
Pre-eclampsia and eclampsia	1.97 (1.0-3.6)	1.7 (0.8-3.3)	1.95 (1-3.6)	1.69 (0.8-3.3)
Small for gestational age	1.89 (1.0-3.7)	2.0 (1.4-2.0)	1.92 (1-3.8)	2.1 (1.0-4.3)
Premature rupture of the amniotic membranes	5.1 (0.5-44.1)	2.6 (0.2-24.7)	5.3 (0.6-46.7)	2.9 (0.3-27.3)
Excessive bleeding after delivery	0.74 (0.25-2.1)	0.9 (0.3-2.9)	0.78 (0.26-2.3)	1.0 (0.3-3.2)
Low birth weight	3.0 (0.3-29.4)	3.8 (0.3-38.6)	3.0 (0.3-29.7)	3.8 (0.3-38.6)

* CI: confidence interval

According to the results of this study it seems that performance cautious and preventive measures about having long working hours and rotating shift work, especially in late pregnancy are necessary. A clear designed work plan for pregnant women and women planning to become pregnant is useful.

This study had some limitations: the study was cross-sectional, so finding a causal relationship was not possible. The study suffered from a recall bias which could not be controlled.

## Conclusion

The results of this study showed that working in a rapid cycling schedule of shift work may cause an increase in the incidence of preterm delivery in pregnant mothers.
